# Quality Characteristics, Sensory Profiles and Ethylene Production of Stored ‘Abate Fetel’ Pears Sorted at Harvest by Time-Resolved Reflectance Spectroscopy

**DOI:** 10.3390/plants12234013

**Published:** 2023-11-29

**Authors:** Maristella Vanoli, Maurizio Grassi, Marina Buccheri, Giovanna Cortellino, Fabio Lovati, Rosita Caramanico, Pietro Levoni, Alberto Dalla Mora, Lorenzo Spinelli, Alessandro Torricelli

**Affiliations:** 1 Consiglio per la Ricerca in Agricoltura e L’analisi Dell’economia Agraria (CREA), Centro di Ricerca Ingegneria e Trasformazioni Agroalimentari, Via Venezian 26, 20133 Milan, Italy; venini61@hotmail.com (M.G.); marina.buccheri@crea.gov.it (M.B.); giovanna.cortellino@crea.gov.it (G.C.); fabio.lovati@crea.gov.it (F.L.); rosita.caramanico@crea.gov.it (R.C.); 2 Dipartimento di Fisica, Politecnico di Milano, Piazza Leonardo da Vinci 32, 20113 Milan, Italy; pietro.levoni@polimi.it (P.L.); alberto.dallamora@polimi.it (A.D.M.); alessandro.torricelli@polimi.it (A.T.); 3 Consiglio Nazionale delle Ricerche, Istituto di Fotonica e Nanotecnologie, Piazza Leonardo da Vinci 32, 20133 Milan, Italy; lorenzo.spinelli@polimi.it

**Keywords:** nondestructive technique, TRS, maturity, controlled atmosphere, 1-MCP, texture, soluble solids, acidity

## Abstract

Time-resolved reflectance spectroscopy (TRS), a nondestructive technique, can help the industry to provide high-quality fruit to encourage pear consumption. The absorption coefficient measured by TRS at 670 nm (*μ*_a_670) represents a maturity index for pear fruit, with less mature pears high *μ*_a_670 and more mature low *μ*_a_670. The aim of this work was to study the quality characteristics, the sensory profiles and the ethylene production of ‘Abate Fetel’ pears sorted at harvest in different TRS maturity classes and stored in different atmospheres. At harvest, 540 pears were measured by TRS for *μ*_a_670, ranked by *μ*_a_670 in three maturity classes (less-LeM, medium-MeM and more-MoM mature) and randomized in nine samples according to 1-MCP treatment (treated, control), storage time (4–6 months) and atmosphere (air-NA; CA: 8–12 kPa O_2_, 1 kPa CO_2_). Fruits were examined at harvest and after 7 days of poststorage shelf life for skin color, firmness, soluble solids, acidity and ethylene production and were submitted to sensory analysis. At harvest and after storage, MoM pears were less green and showed a higher SSC content than LeM ones. After storage, MoM pears produced less ethylene and were perceived to be firmer (especially in 1-MCP-treated pears), more astringent and less juicy (when stored for 6 months) than LeM ones.

## 1. Introduction

The ‘Abate Fetel’ pear, the most important winter pear cultivar in Italy, is appreciated by consumers for its aroma, texture and balanced sweet and sour taste [1,2]. ‘Abate Fetel’ pears are usually stored in air (normal atmosphere-NA) at −0.5/−1 °C for no longer than 4 months, when the fruit becomes sensitive to superficial scald and can lose their ripening ability, remaining firm and dry [1,3,4]. A controlled atmosphere (CA) has been adopted to extend the storage life of ‘Abate Fetel’ pears because it prevents the development of superficial scald; however, the low oxygen levels used in a CA can induce soft scald [4,5,6]. In order to reduce the incidence of soft scald, ‘Abate Fetel’ pears are kept in high O_2_ CA with 5–8 kPa O_2_ and 1–2 kPa CO_2_, even if at these oxygen concentrations storability is only slightly extended [2,7]. The 1-methylcyclopropene (1-MCP) treatment at harvest in combination with NA or high O_2_ CA, and with a storage temperature of 1 °C, prevented the development of superficial scald and soft scald. Furthermore, this storage protocol prolonged the storage duration up to 27–35 weeks and, at the same, allowed the fruits to ripen [2]. Generally, consumers like juicy, sweet, melting and buttery pears with aromas typical of the cultivar, while pears with firm–grainy textures and lack of flavor are disliked [3,8,9,10]. A lack of flavor is probably the main cause of the decline in pear consumption observed in the last 5 years in Europe and in Italy [10,11,12]. Therefore, the industry needs to provide high-quality fruits to encourage pear consumption. Nondestructive techniques can help producers to harvest fruits at the proper maturity stage to implement the best postharvest strategies to fulfil consumer expectations and to minimize losses along the supply chain. Visible and near-infrared (Vis–NIR) spectroscopy is the most advanced nondestructive technique for fruit quality assessment due to its high repeatability, ease of operation, lack of pollution and measurement stability [13]. NIR, Vis–NIR spectroscopy and hyperspectral imaging have been used to assess soluble solid content, titratable acidity, dry matter and firmness in pears with less or more successful results [14]. Spectroscopic techniques were also used to detect internal browning [15,16,17], to classify pears in different ripening degrees [18] or to predict postharvest shelf-storage time [19]. However, the problem of model robustness is very persistent in pears [20]. An improvement with respect to the standard Vis–NIR approach could be the direct nondestructive measure of the relevant quality parameters in the fruit mesocarp, which can be achieved by time-resolved reflectance spectroscopy (TRS). TRS is gaining increasing interest in assessing fruit quality due to its accuracy in measuring optical properties in the deep tissues of different horticultural products [21]. In the TRS technique, a short pulse of monochromatic light is injected into the fruit: whenever a photon strikes a scattering center, it changes its trajectory and keeps on propagating in the tissue, until it is eventually re-emitted across the boundary or captured by an absorbing center. Usually, the laser light is injected into and collected from the fruits by using two optical fibers placed in contact with the surface at a distance of 1–2 cm. The laser light probes a banana-shaped volume of tissue to a depth of 1–2 cm, in contrast to continuous-wave Vis–NIR spectrophotometers, which have a useful penetration depth of a few millimeters [22] and measure the optical properties of the fruit mesocarp with no or limited influence from the skin [23,24,25,26]. By measuring the photon distribution of time-of-flight, both the absorption (*μ*_a_) and the reduced scattering (*μ*_s_′) coefficients are estimated [23,25]. The absorption coefficients are linked to the presence of chemical compounds (pigments, water), while the scattering coefficients are related to the fruit structure. TRS has been applied mainly in postharvest studies on fruits and vegetables to estimate the internal attributes related to maturity and ripeness, to detect internal defects and to discriminate fruits with different sensory characteristics [21,26]. The *μ*_a_ measured by TRS at 670 nm (*μ*_a_670) is linked to the biological age of a fruit and represents an effective maturity index for different fruits, such as peaches, nectarines, apples, plums, pears and mangoes [26,27]. The *μ*_a_670 declines during fruit growth and ripening as well as during storage and shelf life and can be used to segregate fruits into uniform groups based on the maturity degree [16,26,28,29]. Fruit maturity on trees is not homogenous due to variations in flowering time, fruit position, microclimate and nutritional and hormonal status. This variability is a disadvantage for the fruit industry but can be managed in order to determine the best storage and marketing strategies. Typically, less mature (LeM) fruits have high values of *μ*_a_670, while more mature (MoM) fruits have low values of *μ*_a_670 [26]. Fruits classified at harvest according to TRS showed different quality attributes and sensory profiles at harvest, during shelf life and after storage in different conditions. After cold storage, MoM nectarines showed lower firmness and produced more ethylene and higher total volatiles than LeM fruit, confirming that the classification of fruit at harvest based on *μ*_a_670 can distinguish unripe from ripe fruit [30]. Similarly, cold-stored LeM apples were judged less sweet, aromatic and pleasant than MoM ones [26]. The classification by TRS at harvest strongly interacts with 1-MCP treatment and storage conditions: MoM pears developed graininess when stored in NA, whereas they showed well-balanced sensory characteristics when stored in CA and in DCA, becoming soft, juicy, sweet and sour enough [31].

Thus, the aim of this work was to study the quality characteristics, the sensory profiles and the ethylene production of ‘Abate Fetel’ pears treated with 1-MCP and stored for 4–6 months in different atmospheres in relation to maturity at harvest nondestructively measured by TRS.

## 2. Results

### 2.1. At Harvest

The *μ*_a_670 at harvest ranged from 0.054 to 1.000 cm^−1^ and significantly decreased from LeM pears to MoM ones (Table 1). Skin color (*I*_AD_), texture properties and starch hydrolysis were significantly higher in LeM fruits than in MoM ones, while RSR increased with maturity degree (Table 1). Skin *h°* and TA were not affected by TRS maturity class (Table 1). On average, data on the quality characteristics indicate that pears are suitable for medium–long storage [4,32].

### 2.2. After Storage

#### 2.2.1. *μ*_a_670

**Figure 1:**
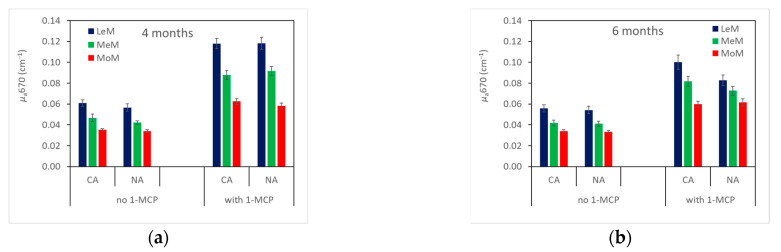
Absorption coefficient measured by TRS at 670 nm (*µ*_a_670) after 4 (**a**) and 6 months (**b**) of storage in ‘Abate Fetel’ pears in relation to 1-MCP treatment, storage atmosphere (CA, controlled atmosphere; NA, normal atmosphere) and TRS maturity class (LeM = less, MeM = medium, MoM = more mature). Bars refer to the standard error of the mean.

The *μ*_a_670 strongly decreased during storage, from 0.176 ± 0.019 (mean ± standard error) at harvest to 0.064 ± 0.001 cm^−1^ after storage plus 7 days of shelf life at 20 °C. The *μ*_a_670 significantly changed with storage time (*F*-ratio = 25.16, *p* < 0.001), 1-MCP treatment (*F*-ratio = 612.77, *p* < 0.001), storage atmosphere (*F*-ratio = 4.18, *p* < 0.05) and TRS maturity class (*F*-ratio = 155.87, *p* < 0.001). The *μ*_a_670 was significantly higher in 1-MCP-treated pears and in LeM fruits and decreased with storage time only in LeM-1-MCP-treated fruits stored in NA and in CA and in MeM-treated fruits stored in NA (Figure 1). Slight differences in *μ*_a_670 were observed according to storage atmospheres, showing 1-MCP NA pears stored for 6 months with lower *μ*_a_670 values than CA ones (Figure 1).

#### 2.2.2. Quality Characteristics

All quality parameters were mostly influenced by 1-MCP treatment and, secondly, by storage time, while storage atmosphere affected only skin color, TRS maturity class skin color and SSC (Table 2).

**Figure 2:**
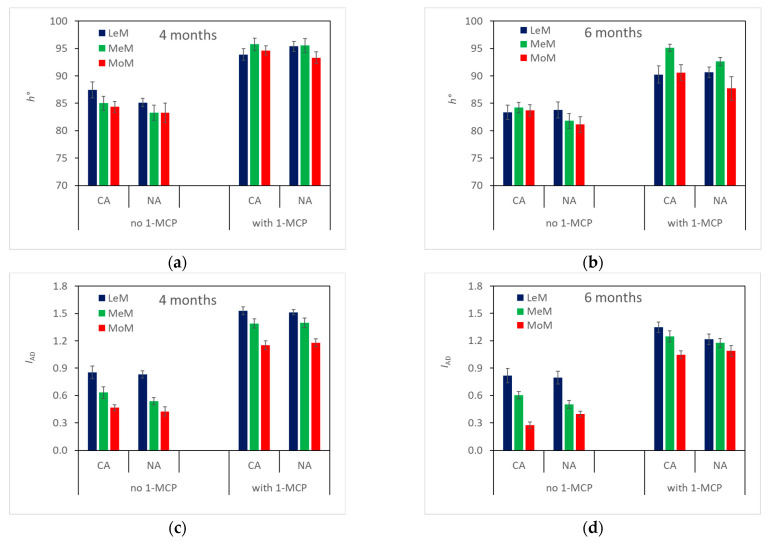
Skin color (*h°*, **top**) and *I*_AD_ (**bottom**) of ‘Abate Fetel’ pears after 4 (**a**,**c**) and 6 months (**b**,**d**) of storage in relation to 1-MCP treatment, storage atmosphere (CA, controlled atmosphere; NA, normal atmosphere) and TRS maturity class (LeM = less, MeM = medium, MoM = more mature). Bars refer to the standard error of the mean.

On average, *h°* (Table 2, Figure 2, top) was lower in control fruits than in 1-MCP-treated ones and was higher in CA fruits than in NA ones. No difference was found among TRS maturity classes except for 1-MCP-treated fruits stored for 6 months, which showed the highest *h°* in MeM-CA pears and the lowest in MoM-NA pears. *h°* significantly decreased from 4 to 6 months of storage, indicating that skin color turned from green to pale green–yellow during storage. 

The *I*_AD_ index (Table 2, Figure 2), on average, was significantly higher in 1-MCP-treated fruits than in control ones and showed the highest values in LeM fruits and the lowest in MoM ones, with no difference between storage atmospheres. During storage, the *I*_AD_ index decreased from 4 to 6 months of storage, mainly in MoM control pears stored in CA and, considering 1-MCP fruit, in LeM fruits stored both in CA and in NA and in MeM fruits stored in NA.

**Figure 3:**
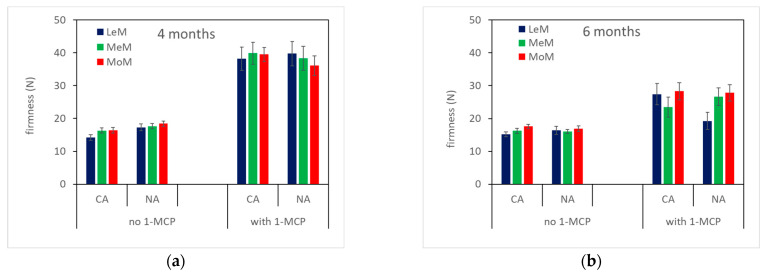

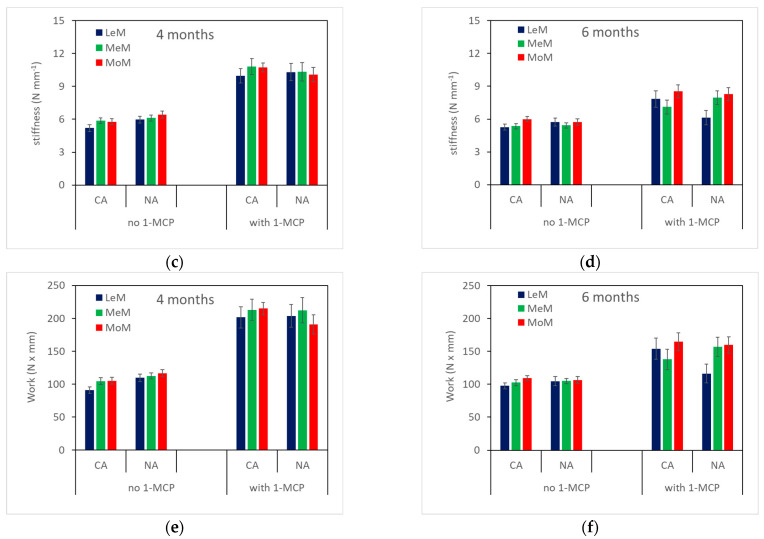
Texture properties (firmness, top; stiffness, center; work, bottom) of ‘Abate Fetel’ pears after 4 (**a**,**c**,**e**) and 6 months (**b**,**d**,**f**) of storage in relation to 1-MCP treatment, storage atmosphere (CA, controlled atmosphere; NA, normal atmosphere) and TRS maturity class (LeM = less, MeM = medium, MoM = more mature). Bars refer to the standard error of the mean.

As for texture (Table 2, Figure 3), all mechanical parameters were strongly affected by 1-MCP treatment, as firmness, stiffness and work showed higher values in treated fruits than in control ones. Storage time affected only 1-MCP pears, as firmness, stiffness and work significantly decreased in these fruits from 4 to 6 months of storage, while no difference was found in control ones. Storage atmosphere and TRS maturity class did not influence texture.

**Figure 4:**
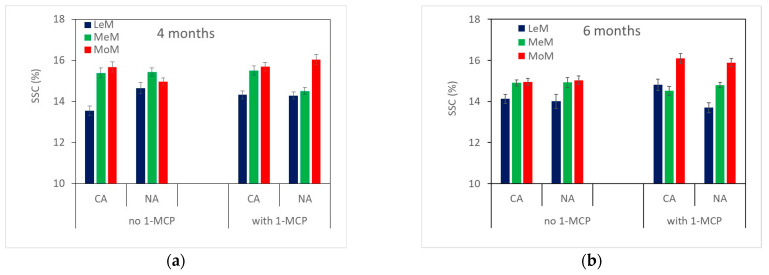

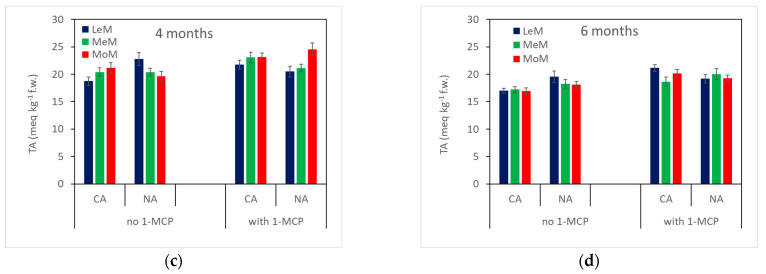
Soluble solids content (**top**) and titratable acidity (**bottom**) of ‘Abate Fetel’ pears after 4 (**a**,**c**) and 6 months (**b**,**d**) of storage in relation to 1-MCP treatment, storage atmosphere (CA, controlled atmosphere; NA, normal atmosphere) and TRS maturity class (LeM = less, MeM = medium, MoM = more mature). Bars refer to the standard error of the mean.

The soluble solids content (Table 2, Figure 4) showed the highest values in MoM fruits and the lowest in LeM ones, except for control fruits stored in NA for 4 months, which exhibited similar values for the three maturity classes. When pears were stored in CA, SSC was higher in 1-MCP fruits than in control ones. However, no differences in SSC were observed between treated and untreated fruits when stored in NA. SSC decreased with storage time only in MoM control pears and in MeM-treated pears stored in CA, while no significant difference was observed for the storage atmosphere.

Titratable acidity (Table 2, Figure 4), on average, showed higher values in 1-MCP-treated pears than in control ones and significantly decreased with storage time, while it did not change with storage atmosphere and TRS maturity class.

#### 2.2.3. Ethylene Production

**Figure 5:**
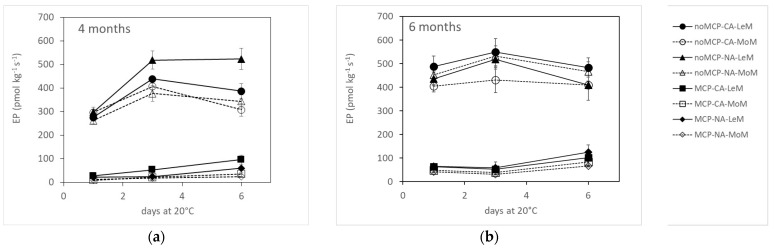
Ethylene production of ‘Abate Fetel’ pears after 4 (**a**) and 6 months (**b**) of storage in relation to 1-MCP treatment, storage atmosphere (CA, controlled atmosphere; NA, normal atmosphere) and TRS maturity class (LeM = less, MeM = medium, MoM = more mature). Bars refer to the standard error of the mean.

Ethylene production during shelf life at 20 °C was mainly affected by 1-MCP treatment (*F*-ratio = 1564.23, *p* < 0.001), secondly by storage time (*F*-ratio = 45.11, *p* < 0.001) and then by TRS maturity class (*F*-ratio = 18.32, *p* < 0.001) and day of shelf life (*F*-ratio = 13.02, *p* < 0.001). On average, 1-MCP-treated pears developed (mean ± standard error) 47 ± 4 pmol kg^−1^s^−1^ and control fruits 417 ± 10 pmol kg^−1^s^−1^. Ethylene production was significantly higher in LeM pears (300 ± 21 pmol kg^−1^s^−1^) than in MoM ones (223 ± 19 pmol kg^−1^s^−1^) and increased from 4 (226 ± 18 pmol kg^−1^s^−1^) to 6 months of storage (295 ± 22 pmol kg^−1^s^−1^). In control fruits stored for 4 months, ethylene showed the highest production after 3 days of shelf life in MoM pears, while in LeM fruits, ethylene increased from 1 to 3 days, maintaining this level until the end of the shelf-life period (Figure 5a). In 1-MCP-treated fruits stored for 4 months, ethylene production did not significantly change with shelf life in NA-stored pears, whatever the TRS maturity class, while in CA-stored pears, it increased from 1 to 6 days of shelf life in LeM fruits, while in MoM fruits, it remained constant up to 3 days and then increased up to 6 days at 20 °C (Figure 5a). No significant difference in ethylene production was observed during shelf life in pears stored for 6 months, regardless of 1-MCP treatment, storage atmosphere and TRS maturity class (Figure 5b).

#### 2.2.4. Sensory Profiles

**Figure 6:**
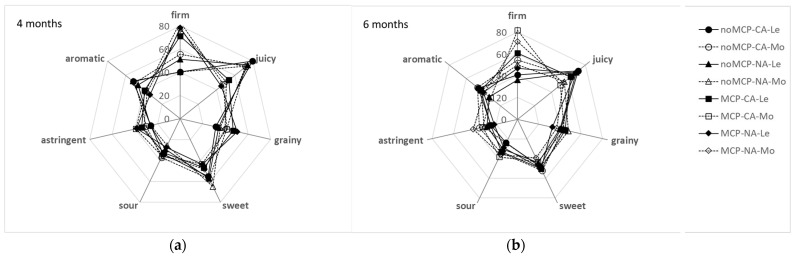
Sensory profiles of ‘Abate Fetel’ pears after 4 (**a**) and 6 months (**b**) of storage in relation to 1-MCP treatment, storage atmosphere (CA, controlled atmosphere; NA, normal atmosphere) and TRS maturity class (LeM = less, MeM = medium, MoM = more mature).

The sensory profiles of ‘Abate Fetel’ pears stored for 4 and 6 months in CA and in NA (Figure 6) were mainly affected by 1-MCP treatment, with control fruits being less firm (*F*-ratio = 39.14, *p* < 0.001), more juicy (*F*-ratio = 18.24, *p* < 0.001), sweeter (*F*-ratio = 8.50, *p* < 0.01) and aromatic (*F*-ratio = 10.08, *p* < 0.01) than 1-MCP-treated ones. 

TRS maturity class also significantly influenced sensory firmness (*F*-ratio = 7.63, *p* < 0.01), juiciness (*F*-ratio = 3.97, *p* < 0.05) and astringency (*F*-ratio = 4.93, *p* < 0.05), with MoM pears being firmer (especially in 1-MCP-treated pears), more astringent and less juicy than LeM ones, especially when stored for 6 months (Figure 6). Storage atmosphere affected only sourness (*F*-ratio = 4.25, *p* < 0.05) with pears stored in NA being less sour than those stored in CA (Figure 6). During storage, sensory firmness, sourness and astringency did not change, regardless of 1-MCP treatment; sweetness and aroma decreased from 4 to 6 months of storage in control pears, while they did not change in 1-MCP-treated fruits; and juiciness increased with storage time in 1-MCP-treated pears and decreased in control ones, and the opposite was observed for graininess (Figure 6). 

**Figure 7:**
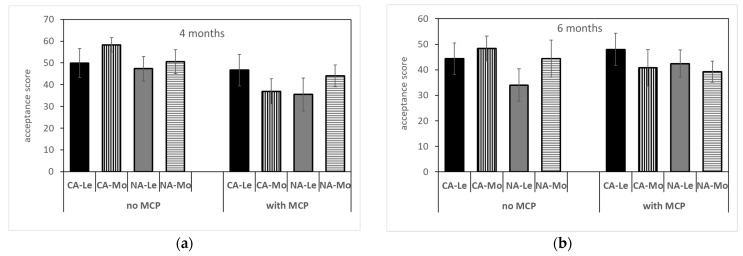
Overall acceptability of ‘Abate Fetel’ pears after 4 (**a**) and 6 months (**b**) of storage in relation to 1-MCP treatment, storage atmosphere (CA, controlled atmosphere; NA, normal atmosphere) and TRS maturity class (LeM = less, MeM = medium, MoM = more mature). Bars refer to the standard error of the mean.

Overall acceptability (Figure 7) did not significantly change with storage conditions, 1-MCP treatment and TRS maturity class, even if it showed, on average, higher scores for control pears stored for 4 months, in both the CA and in the MoM ones. 

#### 2.2.5. PCA Analysis

**Figure 8:**
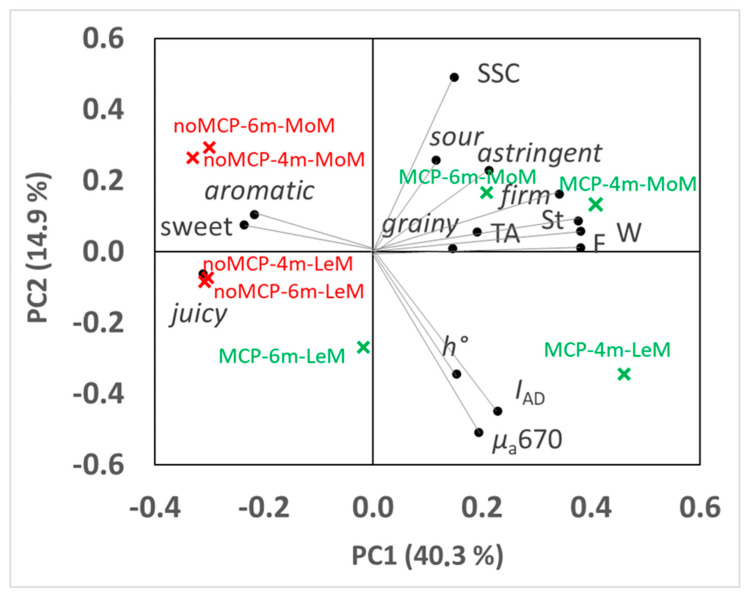
PCA analysis on sensory scores, quality parameters and *µ*_a_670 of ‘Abate Fetel’ pears in relation to 1-MCP treatment (noMCP, MCP), storage time (4 m = 4 months; 6 m = 6 months) and TRS maturity class (LeM = less, MoM = more mature). Biplot of PC1 vs. PC2.

PCA was carried out to study the relationship among *µ*_a_670, sensory attributes and quality characteristics. The two components, PC1 and PC2, accounted for 40.26% and 14.94% of total variance, respectively (Figure 8). In PC1 firmness, work and stiffness were positively related to sensory firmness and negatively to juiciness, sweetness and aroma. In PC2, soluble solids were inversely related to *µ*_a_670, *I*_AD_ and *h°* (Figure 8). 

The relationships among variables highlighted by PCA were studied and confirmed by Pearson’s correlation analysis (Table 3). In fact, *µ*_a_670 was positively related to *I*_AD_ (*r* = 0.83), *h°* (*r* = 0.42) and firmness (*r* = 0.44). *I*_AD_ was positively related to *h°* (*r* = 0.58) and firmness (*r* = 0.48). Firmness, work and stiffness were positively related to sensory firmness, with *r* ranging from 0.75 to 0.80, and for astringency, *r* = 0.43, and they were negatively related to juiciness and sweetness, with *r* ranging from −0.64 to −0.66 and from −0.40 to −0.42, respectively. SSC was positively related to stiffness (*r* = 0.45) and work (*r* = 0.40) and to sensory firmness, with *r* = 0.45. No significant correlation with r ≥ 0.4 was observed for TA. As for sensory attributes, firmness was negatively related to juiciness (*r* = −0.65) and sweetness (*r* = −0.43) and positively related to astringency (*r* = 0.46); juiciness was positively related to sweetness and aroma with *r* = 0.61 and *r* = 0.62, respectively; and sweetness was positively related to aroma (*r* = 0.68). Overall acceptability was positively related to aroma (*r* = 0.83), sweetness (*r* = 0.71) and juiciness (*r* = 0.55).

As for the PC scores, on average, PC1 scores were higher for 1-MCP-treated fruits and for those stored for 4 months, and they did not change with the storage atmosphere or TRS maturity class. PC2 scores were higher for control fruits and for those classified at harvest as MoM by TRS and were not affected by storage conditions. PC1 allowed to discriminate LeM- and MoM-1-MCP-treated pears stored for 4 months (highest PC1 scores) from MoM-1-MCP-treated pears stored for 6 months (intermediate PC1 scores) and LeM-1-MCP-treated pears stored for 6 months (low PC1 scores) and untreated fruits (with any storage time and TRS maturity class), which showed the lowest PC1 scores (Figure 8). PC2 distinguished LeM pears from MoM ones, regardless of 1-MCP treatment and storage time, showing MoM control fruits with the highest PC2 scores and LeM 1-MCP-treated pears with the lowest PC2 scores (Figure 8).

## 3. Discussion

The *µ*_a_670 decreased from harvest to storage plus 7 days of shelf life at 20 °C, indicating that ‘Abate Fetel’ pears ripen during storage time (Table 1, Figure 1). In fact, fruits became pale green–yellow due to the breakdown of chlorophyll content in the skin, as *I*_AD_ significantly decreased (Table 1, Figure 2). At the same time, flesh firmness and acidity decreased, and soluble solids content increased (Table 1, Figure 3 and Figure 4). In ‘Pink Lady^®^’ apples [33], *μ*_a_670 had already decreased after 1 month of cold storage, reflecting the fruits ripening as they lost firmness and hydrolyzed starch. Similarly, in ‘Braeburn’ apples [34], *µ*_a_670 decreased after 3 and 7 months of CA storage, along with changes in the fruits’ texture that were also perceivable by sensory analysis. Rizzolo et al. [30] found that *µ*_a_670 also decreased in peaches stored for different weeks at 1 and 4 °C and that this trend was accompanied not only by fruit softening but also by changes in sugar and acid composition and in volatiles production.

The behavior of *µ*_a_670 of ‘Abate Fetel’ pears during storage was strongly influenced by 1-MCP treatment and secondly by storage conditions (atmosphere, time). In fact, *µ*_a_670 was higher in 1-MCP-treated fruits that were greener, firmer and contained more acid, and they developed much less ethylene than untreated pears (Figure 1, Figure 2, Figure 3, Figure 4 and Figure 5), as previously found by Eccher Zerbini et al. [35], Calvo et al. [36], Rizzolo et al. [3], Folchi et al. [37] and Vanoli et al. [4]. These data also confirm that high *µ*_a_670 values correspond to less ripe fruit. 

In pears, texture is a critical feature of eating quality, and often 1-MCP-treated pears were too hard to be edible no matter the storage protocol, showing firmness values above the threshold of 40 N, corresponding to the upper limit for edible–firm ‘Abate Fetel’ pears [1]. In this work, 1-MCP-treated pears showed, on average, a firmness of about 38.7 N after 4 months of storage and of 25.4 N after 6 months of storage. These values correspond to firm and medium–soft fruit, respectively, according to Predieri and Gatti [1], Rizzolo et al. [3] and Vanoli et al. [4]. This is in contrast with previous findings on 1-MCP-treated pears, which showed firmness values higher than 40 N after 6 months of storage in NA, CA and DCA at −0.5 °C plus 7 days of shelf life at 20 °C [2,3,4,35,36]. On the other hand, Folchi et al. [37] found that early-harvested ‘Abate Fetel’ (68.6 N) after 17 and 34 weeks of NA storage showed firmness values of 54.9 and 45.1 after 7 days at 20 °C, respectively, while pears picked at an optimal harvest date (58.8 N) had firmness values of 36.3 and 30.4, respectively. Similarly, Chiriboga et al. [38] reported that ‘Conference’ pears treated with 1-MCP before or around the commercial harvest date lost their ability to soften even after several days at 20 °C, while in pears treated at more advanced maturity, softening was slowed down but not completely blocked. Untreated fruits showed, on average, a firmness of about 16,6 N (Figure 3), whatever the storage time, which is typical of soft fruit, as previously observed by other authors [1,3,4,35,36,37]. From sensory analysis, 1-MCP-treated fruits were perceived as firmer and less sweet, juicy and aromatic than control ones, whatever the storage time and the storage conditions (Figure 6), in agreement with other authors [3,39,40]. However, no differences were found between treated and untreated pears in overall acceptability, even if control pears stored for 4 months showed the highest acceptability due to the highest scores of juiciness, sweetness and aroma and the lowest scores of graininess (Figure 6 and Figure 7). In our work, as expected, acceptability was positively related to aroma, sweetness and juiciness (Table 3) [3,8].

The *µ*_a_670 was also slightly higher in CA-stored pears than in NA ones (Figure 1), with CA pears showing greener skin (Figure 2), even if the other quality and sensory characteristics and the ethylene production did not change with the atmosphere (Figure 3, Figure 4, Figure 5 and Figure 6). On the contrary, other authors [3,4,35] found that ‘Abate Fetel’ pears stored in NA softened less than CA-stored fruit, even if they were less green and developed more ethylene than CA ones and were less appreciated by consumers as the fruits became firmer and grainier and less sweet, juicy and aromatic [1,3,8]. Folchi et al. [7] found that ‘Abate Fetel’ pears stored in NA did not show consistent differences in texture with CA ones, and that after 180 days of storage, CA pears were sufficiently firm to be marketed, while NA ones were too soft. These contrasting results could be due to the fact that in the present work, O_2_ was set at 8–12%, a percentage which controls the development of superficial scald and prevents the onset of soft scald (a physiological disorder typical of ‘Abate Fetel’ pears stored under 6% of O_2_). In the previous works, O_2_ was set instead at 2%, a percentage that keeps the pears in a less advanced maturity state and inhibits superficial scald but can promote soft scald development [4,7]. Rizzolo et al. [2] found that 1-MCP pears stored in CA with 8% O_2_ were greener and firmer than NA ones after 27 and 35 weeks of storage, while no influence on firmness was observed after 18 weeks. The authors also found that at 18 weeks, CA-stored pears were juicier, more grainy and sourer than NA fruit, while at 27 weeks, they were juicier than CA + NA ones, and after 35 weeks, they were less firm and aromatic than NA ones. The storage atmosphere did not have any significant effect on overall acceptability, no matter the storage time. Also in this case, the different results could be partially explained by the different adopted O_2_ percentages.

Storage time also affected *µ*_a_670 in combination with 1-MCP treatment, as in the control fruits, no change in *µ*_a_670 was observed compared between 4 and 6 months of storage, while in 1-MCP-treated pears, *µ*_a_670 decreased with storage time (Figure 1). This trend in *µ*_a_670, again, mirrored the changes in quality characteristics, as, in untreated fruits no difference was observed for skin color, texture or SSC content, while in treated fruit, the ripening process continued during storage, as skin color became less green and IAD, firmness and acidity decreased (Figure 2, Figure 3 and Figure 4). An opposite trend was observed by Rizzolo et al. [2], who found that in 1-MCP-treated ‘Abate Fetel’ pears, softening and yellowing were inhibited in storage and during shelf life, even extending the storage time up to 35 weeks. 

An opposite trend was observed for sensory attributes (Figure 6), as they did not change with storage time in 1-MCP-treated pears, while in the control fruits, juiciness, sweetness and aroma decreased and graininess increased, causing a decrease in overall acceptability (Figure 7) in fruit stored for 6 months. Other authors [1,3,39] reported that storage time has an impact on the sensory characteristics of untreated ‘Abate Fetel’ pears, as sensory firmness and graininess increased and juiciness decreased with storage time, mainly after 7 days at 20 °C in NA stored fruits, with a concomitant decrease in consumer acceptance. Vanoli et al. [39] found that in ‘Abate Fetel’ pears treated with 1-MCP and stored in CA, after 2 weeks of initial low-oxygen stress, sensory firmness, juiciness and sweetness decreased with storage time, while Rizzolo et al. [2] observed an increase in graininess and a decrease in sourness and astringency in treated ‘Abate Fetel’ pears stored in NA and in CA, without affecting overall acceptability.

Sorting fruits at harvest by TRS on a *µ*_a_670 basis allowed the fruits to be divided into different maturity classes, which showed different behaviors at harvest, after storage in different conditions and in combination with 1-MCP treatment. At harvest, MoM pears were less green, less firm and showed a higher SSC content than LeM ones (Table 1), confirming that they were in a slightly advanced ripening state. However, MoM fruits also showed less advanced starch hydrolysis (Table 1), which is typical of unripe fruit. In previous work, early-picked ‘less mature’ apples had a higher protopectin index than ‘more mature’ ones, indicating a less advanced breakdown of insoluble protopectins to soluble pectins, while no difference between maturity classes was observed for later-picked apples [41]. On the other hand, no significant correlations were found between the absorption coefficients measured in the chlorophyll range by TRS and polyuronide or AIS contents; indeed, starch granules could interfere with TRS measurements, especially with scattering coefficients [41]. After storage, the TRS maturity class affected skin color (Figure 2), SSC content (Figure 4), ethylene production (Figure 5) and some sensory attributes (firmness, juiciness, astringency) (Figure 6) in combination with 1-MCP treatment, storage time and storage atmosphere, while it did not influence texture properties (Figure 3), acidity, (Figure 4) or sensory graininess, sweetness, sourness, aroma (Figure 6) or overall acceptability (Figure 7). MoM pears showed lower *I*_AD_ values and higher SSC content than LeM ones, regardless of 1-MCP treatment or storage conditions. This scenario confirms again that MoM pears are actually more mature than LeM fruits. However, this result is not supported by the ethylene production, which was higher in LeM fruits than in MoM fruits, nor by firmness and acidity, which did not change with maturity class. Eccher Zerbini et al. [35] found that control ‘Abate Fetel’ showed a higher climacteric peak in MoM mature fruits than in LeM ones with an earlier ethylene decrease. In pears treated with 1-MCP at 100 ppb, an increasing amount in ethylene production was observed in LeM fruits and a decreasing amount in MoM ones, while with 1-MCP at 300 ppb, no difference between maturity classes was observed. Eccher Zerbini et al. [35] also found that *h°* and firmness were higher, on average, in less mature pears. Folchi et al. [7,37], Calvo et al. [42] and Lindo-Garcia et al. [43] found lower ethylene production in early-picked pears than in late-harvested ones. In nectarines [30], MoM fruits showed lower firmness at harvest, when ethylene production was not different among TRS maturity classes, and also after storage, when LeM fruits produced less ethylene than MoM ones. In fresh-cut apples, the maturity degree assessed at harvest by TRS did not significantly influence the average amount of ethylene production, even if MoM slices reached the maximum ethylene production earlier than LeM ones [44]. A similar trend was observed in this work, as in LeM pears, ethylene production showed an increasing trend with shelf lifetime, while in MoM pears, the climacteric peak was reached after 3 days at 20 °C. The TRS maturity class also affected sensory profiles. It is well known that pear fruits harvested early are incapable of developing acceptable flavor [45] and are the least appreciated by consumers, being less sweet, juicy and aromatic than fruits from subsequent harvest dates [8]. Oppositely, fruits picked too late showed low acceptance by consumers, as they were overripe, had a bad taste, lacked juice and had starchy pulp [46]. Our findings showed that changes in sensory characteristics do not always reflect the results of instrumental analyses. In fact, even if no difference in firmness was observed between LeM and MoM pears, panelists judged MoM pears as firmer than LeM ones when treated with 1-MCP. No significant difference in sweetness was found, even if SSC content was higher in MoM pears than in LeM ones. Probably, the differences in firmness between LeM and MoM, although not significant, were perceived by sensory analysis, while the different values observed for SSC were not sufficient to be perceived by sensory analysis. A high and significant correlation was found between mechanical and sensory firmness, while no correlation was found between SSC and sweetness. Juiciness was lower and astringency was higher in MoM pears than in LeM ones, but only after prolonged storage. In previous work on ‘Abate Fetel’ pears [31], LeM pears showed the lowest scores of firmness, graininess, sweetness, sourness and aromatic, partially confirming our results. Moreover, the TRS maturity class strongly interacted with storage atmospheres, as MoM pears stored in CA and in DCA showed high juiciness, sweetness, sourness, astringency and aroma and low graininess, while MoM pears stored in NA showed the highest scores for firmness and graininess and the lowest for juiciness. In our work, no effect of storage atmospheres on sensory characteristics was observed, and fruits from different maturity classes can be stored both in NA and CA, obtaining very similar sensory profiles. On the contrary, The TRS maturity class interacted with 1-MCP treatment, as no difference regarding the sensory attributes was observed between LeM and MoM control fruits, while in 1-MCP-treated pears, MoM fruits were judged as firmer than LeM ones.

When considering the quality and sensory characteristics together, MoM and LeM pears treated with 1-MCP and stored for 4 months showed the highest values of texture and sensory firmness and the lowest values for aroma, sweetness and juiciness. However, MoM pears also showed the highest SSC content and the lowest IAD values, while the opposite was found for LeM ones. The 1-MCP-treated pears stored for 6 months showed intermediate values of texture and sensory attributes, but MoM fruits had high values of SSC and LeM low ones. Untreated fruits showed the lowest values of texture and sensory firmness and the highest values of juiciness, sweetness and aroma, with MoM fruits having high SSC content and LeM ones having low SSC content, regardless of storage time.

## 4. Materials and Methods

### 4.1. Fruit and Experimental Plan

The experiment was carried out on ‘Abate Fetel’ pears (*Pyrus communis* L.) picked in 2020 at commercial maturity in an orchard of the Protected Geographical Indication (IGP) area located in the Mantova province (Italy). At harvest, 540 pears were individually measured on two sides by TRS for the absorption coefficient at 670 nm (*μ*_a_670). Pears were ranked by decreasing *μ*_a_670 averaged on the two sides, that is, from the least to the most mature. The ranked fruits were grouped by nines, with a total of 60 groups, corresponding to 60 levels of *μ*_a_670, and divided into 60 groups, corresponding to 60 *μ*_a_670 levels. Each fruit from each group was randomly assigned to a different sample. In this way, 9 batches were obtained, each one containing 60 fruits from the whole range of *μ*_a_670. In each batch, according to fruit ranking, the 60 fruits were divided into three TRS maturity classes: less mature (LeM, rank 1–20), medium mature (MeM, rank 21–40) and more mature (MoM, rank 41–60). The nine batches were randomly assigned to harvest, two 1-MCP treatments (treated, untreated), two storage atmospheres (−1 °C; air-NA; CA: 8 kPa O_2_ + 1 kPa CO_2_ up to 4 months of storage, then 12 kPa O_2_ + 1 kPa CO_2_ up to 6 months) and two storage times (4 and 6 months), as reported in Table 4.

After 4 and 6 months of storage at −1 °C plus 7 days at 20 °C, pears were measured by TRS at 670 nm and were analyzed for skin color, texture properties, soluble solids content and titratable acidity. LeM (rank 1–10) and MoM (rank 51–60) pears were submitted to sensory analysis. At 1, 3 and 6 days of shelf life at 20 °C, ethylene production was measured on LeM (rank 1–5) and Mom (rank 1–5) pears.

At harvest, a sample of 60 fruits was analyzed for fruit mass, skin color, flesh firmness, starch hydrolysis, titratable acidity and soluble solids content.

### 4.2. TRS Measurements

#### 4.2.1. TRS Set-Up

**Figure 9:**
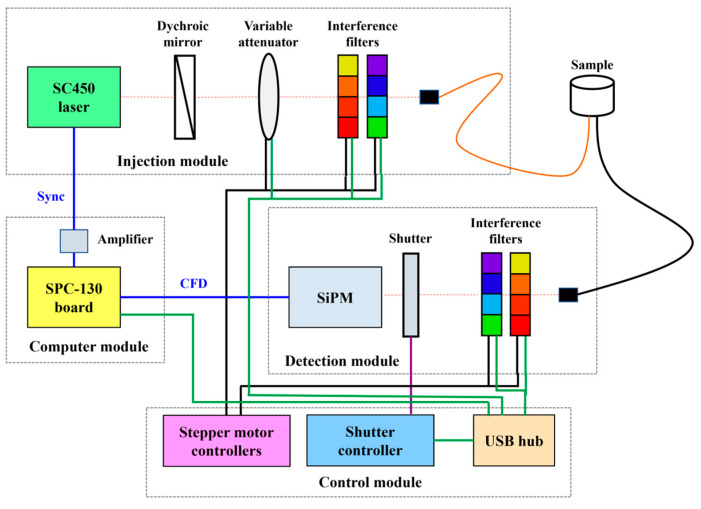
Scheme of the multiwavelength TRS system. Sync: synchronization signal. CFD: constant fraction discrimator. SiPM: silicon photomultiplier module.

An existing instrumentation for TRS developed at the Department of Physics of Politecnico di Milano was used (Figure 9). Briefly, the injection module is based on a high-power supercontinuum fiber laser (SC450-6W, Fianium, UK) emitting white-light picosecond pulses at a repetition rate of 40 MHz. Light attenuation is adjusted via a circular continuously variable neutral density filter (Edmund Optics Inc., Barrington, NJ, USA). A set of 14 bandpass interference filters within the wavelength range 540–1064 nm was employed to select the desired wavelength. The pulse was then coupled to a 200 μm core step-index optical fiber (Thorlabs Inc., Newton, NJ, USA) and delivered to the sample. The detection module is based on a silicon photomultiplier module developed at the Department of Physics of Politecnico di Milano [47]. Before being focused onto the detector, light emitted by the sample is collected by a 1mm core graded-index optical fiber (FiberFin Inc., Yorkville, IL, USA) and filtered by a set of interference filters identical to the one in the injection module. The instrument response function (IRF) of the system is characterized by a full width at a half-maximum of about 100 ps.

#### 4.2.2. TRS Data Analysis

Data acquisition was performed by a Time-Correlated Single-Photon Counting board (Becker&Hickl GmbH, Berlin, Germany) able to reconstruct the distribution of time-of-flight (DTOF) of the detected photons. The DTOF is analyzed via an in-house-built software based on a nonlinear fitting procedure, thus retrieving the values of absorption and reduced scattering coefficients of the sample. The theoretical curves simulating photon diffusion in a turbid homogeneous medium that are required as input of the fitting procedure are calculated by exploiting a model based on the diffusion equation [48].

### 4.3. Quality Parameters

#### 4.3.1. Skin Color

Skin color was measured on two opposite sides of the fruit in L*, a*, b* color space using a Spectrophotometer CM-2600d (Minolta Co., Osaka, Japan), the primary illuminant D65 and a 10° observer. Hue (*h°*) was computed according to h° = arctangent(b*/a*) × 360/ (2 × 3.14). Skin color was also measured through the determination of *I*_AD_ (index of absorbance of chlorophyll) using a DA-meter, a portable nondestructive device based on Vis–NIR spectroscopy (TR, Forlì, Italy). The *I*_AD_ is the difference between the absorbance values at 670 nm and at 720 nm.

#### 4.3.2. Texture Properties

The mechanical properties were determined on two opposite peeled sides of each pear using a penetrometer (TA-XT plus Texture Analyzer, Stable Micro Systems, Godalming, UK; 8 mm diameter plunger; crosshead speed 3.33 mm s^−1^ to a depth of 8 mm). According to Vanoli et al. [28], from the force–displacement curve, three parameters were recorded: flesh firmness (F, maximum force value within 8 mm), stiffness (St, slope of the first part of the force–displacement curve measured from 0 to 2 mm) and work (W, energy related to flesh penetration up to 8 mm). Firmness, stiffness and work data were averaged for each fruit.

#### 4.3.3. Starch Hydrolysis, Soluble Solids Content and Titratable Acidity

The stage of starch hydrolysis was determined on equatorially cut pears soaked into a Lugol solution and by comparing the staining pattern of the pulp with the EUROFRU scale (1–10; 1 = minimum, 10 = maximum starch hydrolysis). Soluble solids content (SSC) and titratable acidity (TA) were determined using juice squeezed from each fruit; SSC was measured by placing some drops of juice on an automatic refractometer (RFM81, Bellingham-Stanley Ltd., England); and TA was determined by titrating 5 g of juice plus 50 mL of distilled water with 0.1 N NaOH to pH 8 using a 682 titroprocessor equipped with a 665 dosimat (Metrohm, Herisau, Switzerland).

### 4.4. Ethylene Production

Ethylene production was measured according to Rizzolo et al. [49] on 5 fruit samples. Pears were put in 1.7 L gastight glass jars (1 fruit per jar); after 2 h at 20 °C, 1 mL of gas taken from the headspace of each jar was injected into a gas chromatograph (DANI Instruments 86.10, Monza, Italy) fitted with a VU65 switching valve equipped with a 1 mL loop, an FID detector and a deactivated aluminum oxide F1 (80–100 mesh) column (1/8 in. × 200 cm, Alltech Italia, Sedriano, MI, Italy) at the following temperatures: 100 °C for injector and column and 225 °C for detector. 

### 4.5. Sensory Analysis

Sensory analyses were carried out on LeM and MoM pears in a sensory lab equipped with six computerized individual booths, according to Rizzolo et al. [3]. A panel of 10 individuals who were familiar with pears and available for sensory analysis was recruited. Before tasting the samples, a brief training was carried out in order to verify and discuss the use of flavor (taste and aroma) and texture attributes. One peeled slice/fruit/TRS maturity class/1-MCP treatment/storage atmosphere, coded with three-digit numbers, was presented to ten short-term-trained panelists in a randomized order within 1 h after cutting to avoid browning. Judges were asked to evaluate the intensity of the following attributes: firmness, graininess, juiciness, sweetness, sourness, astringency and aroma, as well as overall acceptability, using 120 mm unstructured line scales with anchors at 12 mm from the extremes (low, high). Definitions of the sensory attributes are as follows [3]: firmness, the resistance to mastication perceived at the first and successive bites; graininess, the not smooth texture perceived during mastication; juiciness, the textural property giving the sensation of progressive increase in the free fluids in the oral cavity during mastication; sweetness, one of the basic tastes (e.g., sucrose); sourness, one of the basic tastes (e.g., malic acid); astringency, the dry, puckering mouth feel perceived during mastication; and aromatic, retronasal olfactory perception.

### 4.6. Statistical Analysis

Data of *μ*_a_670, ethylene production, and quality and sensory parameters were submitted to multifactor ANOVA considering 1-MCP treatment, storage time, storage atmosphere and TRS maturity class as factors; means were compared by Tukey’s test at *p* ≤ 0.05%. In order to explore the relationship among all variables, all data were submitted to Principal Component Analysis (PCA), and Pearson’s correlation analysis was performed: only significant (*p* < 0.001) correlations with *r* ≥ 0.40 were considered. Prior to statistical analyses, the rating scores of each sensory attribute were standardized by panelists according to Bianchi et al. [50] to remove the variability due to panelists using different parts of the scale. Statistical analyses were performed using the Statgraphics ver. 7 (Manugistic Inc., Rockville, MD, USA) software package.

## 5. Conclusions

In conclusion, our results indicate that classifying pears at harvest in homogeneous maturity classes by using the absorption coefficient nondestructively measured by TRS at 670 nm (*µ*_a_670) allowed to obtain fruits with different quality and sensory characteristics after storage, mainly regarding skin color, soluble solids content and sensory firmness. However, due to the strong effect of 1-MCP treatment and, secondly, of storage duration, quality characteristics and sensory attributes were not always very different between less and more mature pears. Therefore, TRS maturity classification at harvest can give partial but not conclusive indications of the best storage strategies to obtain fruits that can satisfy consumers.

## Figures and Tables

**Table 1:** *μ*_a_670 and quality characteristics (mean ± se) of ‘Abate Fetel’ at harvest according to TRS maturity class (LeM, less mature; MeM, medium mature; MoM, more mature) and ANOVA results.

	LeM	MeM	MoM	Mean	*p*-Value
*μ*_a_670 (cm^−1^)	0.290 ± 0.049	0.137 ± 0.003	0.098 ± 0.003	0.176 ± 0.019	***
Skin color *h°*	103.7 ± 1.5	102.3 ± 1.4	98.7 ± 2.1	101.5 ± 1.0	ns
Skin color *I*_AD_	1.87 ± 0.02	1.81 ± 0.03	1.65 ± 0.05	1.78 ± 0.02	***
Starch hydrolysis	6.6 ± 0.5	4.4 ± 0.4	2.9 ± 0.2	4.6 ± 0.3	***
Firmness (N)	53.2 ± 1.5	49.1 ± 1.4	46.9 ± 1.3	49.7 ± 0.9	**
Stiffness (N mm^−1^)	14.8 ± 0.6	14.5 ± 0.3	14.0 ± 0.2	14.4 ± 0.2	ns
Work (N × mm)	303.0 ± 8.8	286.1 ± 7.4	273.8 ± 6.0	287.7 ± 4.6	*
SSC (%)	13.6 ± 0.2	14.2 ± 0.2	15.0 ± 0.2	14.3 ± 0.1	***
TA (meq kg^−1^)	30.0 ± 1.4	26.1 ± 0.9	28.3 ± 1.4	28.2 ± 0.8	ns

*p*-value of *F* ratio: ns, not significantly different; * *p* < 0.05; ** *p* < 0.01; *** *p* < 0.001.

**Table 2:** Results of multifactorial ANOVA for color parameter (*h°*, *I*_AD_), texture properties (F—firmness; St—Stiffness; W—work), soluble solids content (SSC) and acidity (TA) of ‘Abate Fetel’ pears in relation to storage time, 1-MCP treatment, storage atmosphere and TRS maturity class.

	*h°*	*I*_AD_	F	St	W	SSC	TA
**Main effects**							
Storage time (A)	ns	ns	ns	ns	ns	ns	ns
1-MCP treatment (B)	***	***	ns	**	ns	ns	**
Storage atmosphere (C)	ns	ns	ns	ns	*	ns	ns
TRS maturity class (D)	**	*	ns	ns	ns	*	ns
**Interactions**							
A × B	ns	*	**	*	ns	ns	ns
A × C	ns	ns	ns	ns	ns	ns	ns
A × D	ns	ns	ns	ns	ns	*	ns
B × C	ns	ns	ns	ns	ns	ns	ns
B × D	ns	ns	*	ns	ns	ns	ns
C × D	ns	ns	ns	ns	ns	ns	ns
A × B × C	ns	ns	ns	ns	ns	ns	ns
A × b × D	ns	ns	ns	ns	ns	ns	ns
A × C × D	ns	ns	ns	ns	ns	ns	ns
B × C × D	ns	ns	ns	ns	*	ns	ns
A × B × C × D	ns	ns	ns	ns	ns	ns	ns

*p*-value of *F* ratio: ns, not significantly different; * *p* < 0.05; ** *p* < 0.01; *** *p* < 0.001.

**Table 3:** Pearson’s correlation matrix among *µ*_a_670, quality characteristics (F, firmness; St, stiffness; W, work; SSC, soluble solids content; TA, titratable acidity) and sensory properties (FF, firm; J, juicy; Gr, grainy; Sw, sweet; So, sour; As, astringency; Ar, aroma; Acp, acceptability). Significant correlations are in bold (N = 148; r ≥ 0.4, *p*-value ≤ 0.05).

	*µ*_a_670	*h°*	*I*_AD_	F	St	W	SSC	TA	FF	J	Gr	Sw	So	As	Ar	Acp
*µ*_a_670	**1**															
*h°*	**0.42**	**1**														
*I*_AD_	**0.83**	**0.58**	**1**													
F	**0.44**	0.36	**0.48**	**1**												
St	0.34	0.30	**0.41**	**0.96**	**1**											
W	0.38	0.32	**0.43**	**0.99**	**0.98**	**1**										
SSC	−0.37	−0.08	−0.17	0.36	**0.45**	**0.40**	**1**									
TA	0.12	0.30	0.30	0.39	0.39	0.38	0.30	**1**								
FF	0.22	0.18	0.33	**0.75**	**0.80**	**0.79**	**0.45**	0.39	**1**							
J	−0.23	−0.18	−0.25	**−0.64**	**−0.65**	**−0.66**	−0.28	−0.24	**−0.65**	**1**						
Gr	0.23	0.18	0.17	0.33	0.30	0.31	0.17	0.05	0.26	−0.21	**1**					
Sw	−0.26	−0.08	−0.26	**−0.41**	**−0.40**	**−0.42**	−0.00	−0.19	**−0.43**	**0.61**	−0.12	**1**				
So	−0.05	0.01	0.10	0.19	0.24	0.22	0.32	0.25	0.33	−0.13	−0.05	−0.02	**1**			
As	0.07	−0.04	0.10	**0.43**	**0.43**	**0.44**	0.27	0.17	**0.46**	−0.38	0.24	−0.30	0.35	**1**		
Ar	−0.28	−0.06	−0.28	−0.38	−0.36	−0.38	0.01	−0.08	−0.33	**0.62**	−0.13	**0.68**	−0.01	−0.25	**1**	
Acp	−0.25	−0.06	−0.24	−0.27	−0.25	−0.26	0.12	−0.02	−0.24	**0.55**	−0.12	**0.71**	0.04	−0.30	**0.83**	**1**

**Table 4:** Descriptions of pear batches (NA, air storage; CA, controlled atmosphere).

Batches	Months of Storage	1-MCP	Storage Atmosphere	Storage Temperature
Batch 1	0 (harvest)	-	-	-
Batch 2	4	no	NA	−1 °C
Batch 3	4	no	CA	−1 °C
Batch 4	4	yes	NA	−1 °C
Batch 5	4	yes	CA	−1 °C
Batch 6	6	no	NA	−1 °C
Batch 7	6	no	CA	−1 °C
Batch 8	6	yes	NA	−1 °C
Batch 9	6	yes	CA	−1 °C

## Data Availability

The data from this study are only in this study, there are no archives or databases available elsewhere.
